# AMDPWE: Alphonso Mango Dataset for Precision Weight Estimation

**DOI:** 10.1016/j.dib.2023.109778

**Published:** 2023-11-07

**Authors:** Akshatha Prabhu, N. Shobha Rani

**Affiliations:** Department of Computer Science, School of Computing, Mysuru Campus, Amrita Vishwa Vidyapeetham, India

**Keywords:** Alphonso mangoes, Mass estimation, Computer vision, Mango processing, Sustainable technology

## Abstract

Alphonso Mango (Mangifera indica L.), is popularly known as king of mangoes in India. India is one of the leading countries in mango production. Automatic visual inspection systems for quality assessment using weight are intelligent interventions designed to evaluate fruit maturity based on various parameters. Automated systems utilize a combination of image analysis, computer vision, and artificial intelligence algorithms to estimate the weight of fruits precisely. One of the crucial quality parameters is weight, which measures the fruit's overall mass and potential quality. Integration of precision weighing mechanisms in fruit quality estimation leads to a quick and accurate method of measuring fruit weight in the marketplace. Furthermore, the fruit's demand in the market is directly connected to its size as it influences consumer preferences. Automatic precision weight estimation systems equipped with intelligent high-resolution assists in ensuring consistency in size across batches of fruits.

The dataset samples consist of images of 71 Alphonso cultivars of mango fruit. The fruit is collected from the College of Horticulture Yalachahalli, Mysuru, India. The fruits were harvested in April/May 2022. The digital images of these fruits are captured using the acquisition setup with a controlled environment. Each image has a resolution of 2048×1536. The images include two orientations of each sample. The physical parameters such as the weight, fruit diameter, and width across the shoulder are also maintained. The digital images undergo pre-processing, and further, the vision-based features such as area, convex area, and minor axis for both orientations are captured.

Specifications Table

**Table:** 

Subject	Horticulture, Computer Vision and Pattern Recognition, Agriculture Engineering
Specific subject area	Vision based weight estimation model for Alphonso mangoes
Data format	Raw
Type of data	Images in .JPG format
Data collection	Seventy one samples of Alphonso mangoes were collected in a mango orchard from the College of Horticulture, Yalachahalli, Mysuru, India, with co-ordinates at 12°22′45.6″N 76°31′27.7″E. These were procured in their unripe stage in May /June −2022 with temperature recorded in the 36 °C–38 °C range. The images of the harvested fruit were captured using the acquisition setup. The setup was designed with a sufficient lighting system to capture the images in two orientations and ensure no shadows were formed. Each image is of resolution 2048×1536. The folder size for the dataset is 44.7 MB and is provided for convenient downloading.
Data source location	Institution: College of Horticulture, Yelachahalli, Yelawala, (12°22′45.6″N 76°31′27.7″E) City/Town/Region: Yelachahalli, Yelawala, Mysuru Country: India
Data accessibility	Repository name: Mendeley Data Data identification number: 10.17632/8sjny373pz.1 Direct URL to data: https://data.mendeley.com/datasets/8sjny373pz/1
Related research article	[1] Prabhu, Akshatha, Shobha Rani, N., and Basavaraju, H.T. ‘An Orientation Independent Vision Based Weight Estimation odel for Alphonso Mangoes’. Journal of Intelligent & Fuzzy Systems. Vol. 44, Issue 5, 7257 – 7275.

## 1 Value of the Data

• The dataset of the unripe Alphonso mangoes is available for the public to use and readily available for download. This would assist the researchers in downloading and applying machine learning algorithms directly.
• The physical parameters such as the diameter of the entire fruit, shoulder width, and mango weight that are provided along with the dataset can be used for further postharvest analysis.
• The shape and texture features of the unripe fruit can be extracted from the images, and computer vision systems with algorithms can be applied to determine the stages of ripening, defects, mass and volume estimation on the surface of the fruit [2], [3], [4].
• The data can be used to develop programs, algorithms, and models to assist the people in the food processing line in checking for defects by maintaining quality inspection and control.
• The features extracted from an image can be combined along with parameters such as total soluble solids and fiber, regression techniques, and neural models for inspection and evaluation [5].
• The images captured across orientations provide vision systems to implement non-invasive techniques to assess the quality of the fruit.

## 2 Data Description

The dataset folder contains images of Alphonso fruit. There are 71 samples, each with images of two orientations. Thus, the total number of mango images is 142. Each sample is separated into a folder labeled with a number corresponding to the sample (Table 1).

**Table 1:** Brief description about the data collection.

No	Particulars	Description
1	Fruit	Mango
2	Cultivar	Alphonso
3	Harvest Time	May-June 2022, with temperature ranging from 36 °C – 38 °C
4	Harvest Place	College of Horticulture Yalachahalli, Yelawala, Mysuru,India . co-ordinates at 12°22′45.6″N 76°31′27.7″E
5	Shooting period	May–June 2022, 2 days within the harvest
6	Image size	2048×1536
7	Total samples	71
8	Total images	142

The dataset contains the following information:

1. Sample ID – one for the entire fruit and other two for the two orientations (pertaining to the labeled folders in the ‘Alphonso mangoes image dataset’ folder)
2. Physical parameters - the extracted physical features of the whole fruits, such as fruit diameter (mm), width across the shoulder (mm), and actual weight(gms)

### 2.1 Description of Alphonso mangoes

Alphonso mango cultivated in India, primarily grown in the country's western region, is highly prized due to its taste, aroma, and flavor. Considered the king of mangoes, it is one of the finest varieties. They have oblate shapes ranging from small to medium size with golden yellow skin. Rich in Vitamin C, the pulp has a smooth, creamy texture and is hence used in various savoury dishes. It is harvested in April – June. Its exceptional taste and fruit-keeping quality, and thus, most of it is exported.

### 2.2 Image acquisition

**Fig. 1:**
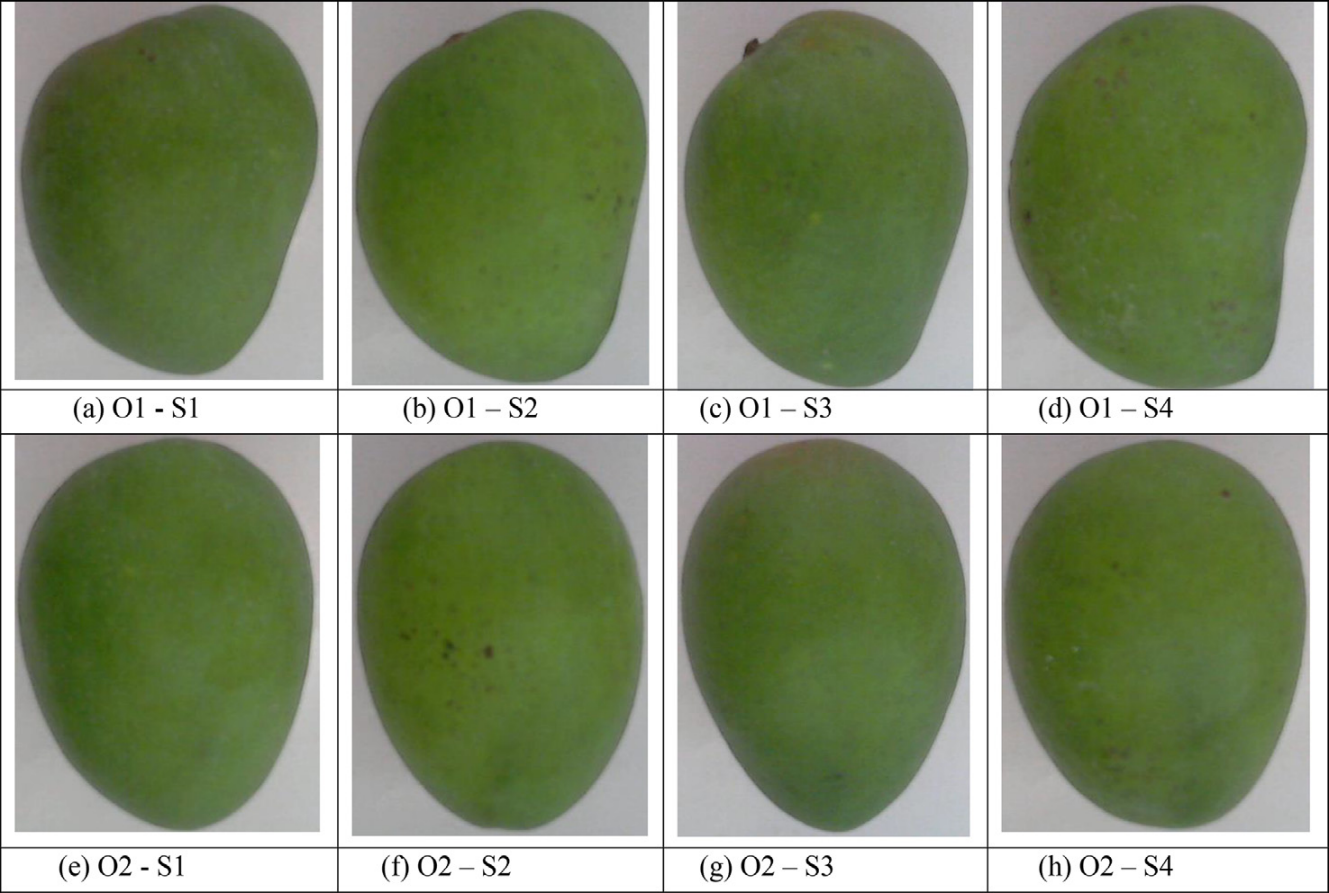
Image samples of mangoes captured in two different orientations S1: Sample 1, S2: Sample 2, S3: Sample 3, S4: Sample 4 (a) O1–S1: Orientation 1 of sample S1 (b) O1–S2: Orientation 1 of sample S2 (c) O1–S3: Orientation 1 of sample S3 (d) O1–S4: Orientation 1 of sample S4 (e) O2–S1: Orientation 2 of sample S1 (f) O2–S2: Orientation 2 of sample S2 (g) O2–S3: Orientation 2 of sample S3 (h) O2–S4: Orientation 2 of sample S4.

The images captured using the setup are shown in Fig 1. S1, S2, S3 and S4 represent samples of the fruit.O1–S1, O1–S2, O1–S3 and O1- S4 represent the Orientation-1 images for samples – S1, S2, S3 and S4 respectively. Similarly, O2–S1, O2–S2, O2–S3, and O2- S4 represent the Orientation- 2 images for samples – S1, S2, S3 and S4, respectively.

## 3 Experimental Design, Materials and Methods

**Fig 2:**
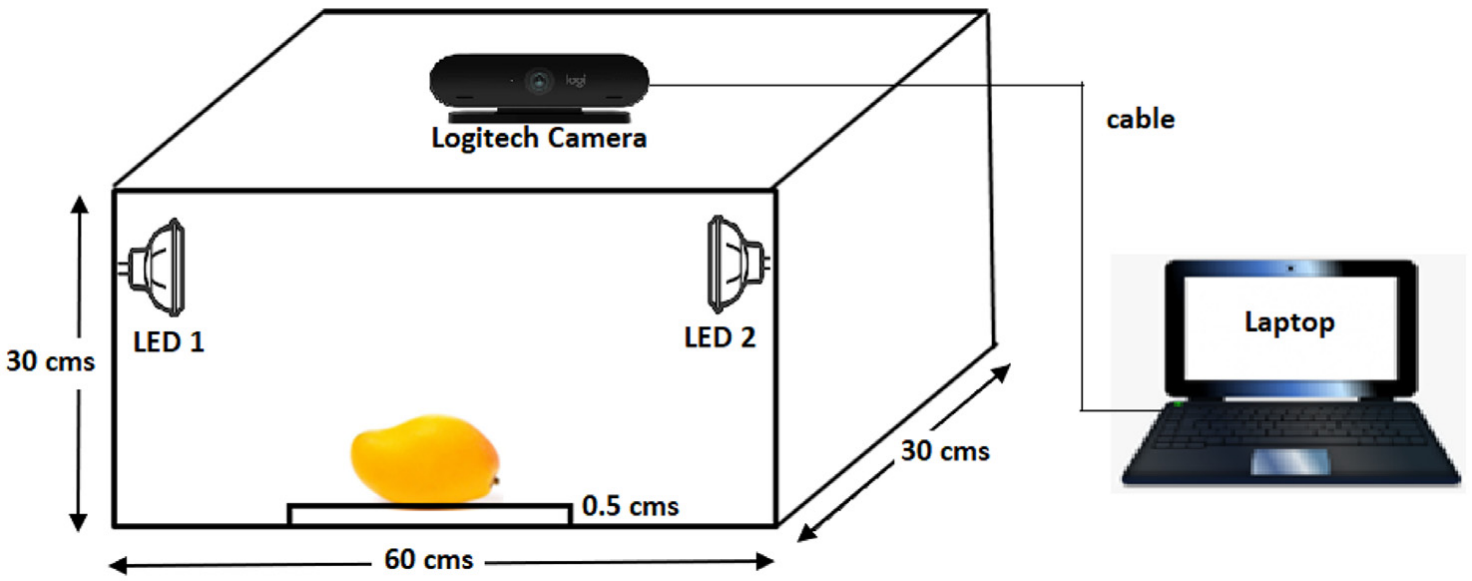
Image acquisition model.

The image acquisition setup was designed to capture the image of the sample across two orientations. The setup designed includes a computer that is connected to the imaging device. The imaging equipment consists of a rectangular wooden case mounted with two LED lamps at slant angles. The base of the rectangular box is flat surface with a length of 30 cm and breadth of 60 cm respectively. The LED lamps that are mounted on the two side walls of a rectangular box act as a source of illumination used to capture the images. The top wall of the rectangular box has an imaging device perpendicularly mounted on it exactly in the middle, 30 cm from the left end and 15 cm from the top of the vertically 30 cm long lid. To firmly hold the sample to the base, inside the setup, a rectangular slab with dimensions of 30 cm in length, 1.8 cm in width, and 0.5 cm is placed. A white sheet of paper of the size of the slab is spread on the surface of it to prevent from being scratched, which happens when the sample is placed directly on the slab. The webcam is installed upside-down through the hole on top of the lid. The image-capturing software is installed in the computer system and it captures the image of the fruit across two orientations .These images are further saved in the folder. The image acquisition setup is shown in Fig 2.

To prevent the reflection on the samples, LED lamps were used to light up a 180◦ region where controlled illumination is used. The bulbs used have the energy efficiency of 60 % to 70 % and are economical as the images were captured over a lengthy period. They also operate on minimum voltage and also compact. To create the base and the wall for the setup, PVC Sunboard Sheets, produced using a foaming agent were used. These sheets are economical, handy to use and provide efficient resistance towards fire, water, and termites.

### 3.1 Camera and imaging software

The Logitech C270 HD Webcam is installed on the upside-down direction of the box through the hole on top of the lid. This camera has fixed focus, automatic light adjustment, a 60-degree diagonal field of vision and 720p/30 fps resolution. The camera has a built-in microphone, an automated noise reduction feature. It supports video calling and can be conveniently used with instant messaging software. The camera wire is plugged in. On the PC, image-capture software is installed to acquire images. The computer system used for image acquisition is a 64-bit operating system, Intel(R) core (TM) i7-4700MQ CPU, 2.40 GHz with 8 GB RAM.

### 3.2 Vernier caliphers and weighing scale

The digital linear Vernier caliper 150 mm (6 “) was used during the collection of the physical measurements of the fruit samples. The following table describes specification of the caliper (Table 2).

**Table 2:** Brief description about the data collection.

No	Specification	Description
1	Measuring Range	0–150 mm/0–6 inch
2	Resolution	0.01 mm/0.0005 inch
3	Repeatability	0.01 mm/0.0005 inch.
4	Maximum measurement speed	1 m/s.
5	Power	1 × 1.5 V LR44 cell (included).

The samples were placed across the outside jaws of the tool. The jaws were adjusted through the thumbwheel that can easily be scrolled in left and right directions. Once the jaws were fixed across the sample, the digital display of the measurements in mm was recorded. Initially, the tips of both ends of the fruit were placed across the caliper's jaws, and the measurements were noted. The fruit was inserted across the two ends of the fully outgrown shoulder in between the outer jaws of the tool and ensured that they were firmly placed. Then, the measurements across the shoulder were documented.

Digital weighing scale-Chef Mate Model KS 33, was used in the work. It has a digital display with a detachable bowl. The machine is powered by two AAA batteries and is built with high-precision sensors. The display is set to 0.0 g, the fruit is placed inside the bowl, and the weight is noted immediately.

## Limitations

For optimal image quality, it's is essential to ensure that fruit has no dirt accumulated on it. Also, the sample must be aligned correctly during image acquisition for test images to achieve accurate results.

## Ethics statement

Alphonso mangoes were collected from the orchard at the University of Horticulture during harvest season. Necessary permissions were obtained during the period. Tools and procedures used for collecting and storing mango samples are referred to as guidelines. As a result, there is no impact on other mango trees. Data acquisition recommendations were taken from experts. Experiments on humans or animals are not involved in this study.

## CRediT authorship contribution statement

**Akshatha Prabhu:** Conceptualization, Software, Resources, Validation, Formal analysis, Writing – original draft, Writing – review & editing, Validation, Data curation. **N. Shobha Rani:** Conceptualization, Software, Resources, Validation, Formal analysis, Writing – original draft, Writing – review & editing, Validation, Data curation.

## Data Availability

AMDPWE: Alphonso Mango Dataset for Precision Weight Estimation (Original data) (Mendeley Data)
